# Sustained Improvements in Glucose Metabolism Late After Roux-En-Y Gastric Bypass Surgery in Patients with and Without Preoperative Diabetes

**DOI:** 10.1038/s41598-019-51516-y

**Published:** 2019-10-22

**Authors:** Nils B. Jørgensen, Kirstine N. Bojsen-Møller, Carsten Dirksen, Christoffer Martinussen, Maria S. Svane, Viggo B. Kristiansen, Jens J. Holst, Sten Madsbad

**Affiliations:** 1 Department of Endocrinology, Hvidovre Hospital, Hvidovre, Denmark; 2 Department of Surgery, Hvidovre Hospital, Hvidovre, Denmark; 3 Faculty of Health and Medical Sciences, University of Copenhagen, Copenhagen, Denmark; 4 Novo Nordisk Center for Basic Metabolic Research, University of Copenhagen, Copenhagen, Denmark

**Keywords:** Type 2 diabetes, Obesity

## Abstract

To describe glucose metabolism in the late, weight stable phase after Roux-en-Y Gastric Bypass (RYGB) in patients with and without preoperative type 2 diabetes we invited 55 RYGB-operated persons from two existing cohorts to participate in a late follow-up study. 44 (24 with normal glucose tolerance (NGT)/20 with type 2 diabetes (T2D) before surgery) accepted the invitation (median follow-up 2.7 [Range 2.2–5.0 years]). Subjects were examined during an oral glucose stimulus and results compared to preoperative and 1-year (1 y) post RYGB results. Glucose tolerance, insulin resistance, beta-cell function and incretin hormone secretion were evaluated. 1 y weight loss was maintained late after surgery. Glycemic control, insulin resistance, beta-cell function and GLP-1 remained improved late after surgery in both groups. In NGT subjects, nadir glucose decreased 1 y after RYGB, but did not change further. In T2D patients, relative change in weight from 1 y to late after RYGB correlated with relative change in fasting glucose and HbA1c, whereas relative changes in glucose-stimulated insulin release correlated inversely with relative changes in postprandial glucose excursions. In NGT subjects, relative changes in postprandial nadir glucose correlated with changes in beta-cell glucose sensitivity. Thus, effects of RYGB on weight and glucose metabolism are maintained late after surgery in patients with and without preoperative T2D. Weight loss and improved beta-cell function both contribute to maintenance of long-term glycemic control in patients with type 2 diabetes, and increased glucose stimulated insulin secretion may contribute to postprandial hypoglycemia in NGT subjects.

## Introduction

Roux-en-Y gastric bypass surgery (RYGB) is a highly effective obesity treatment, resulting in 30–40% weight loss after 1 year^1^. Additionally, it is the most efficient treatment of type 2 diabetes (T2D) in morbidly obese individuals, improving glucose tolerance profoundly within days in the majority of patients and providing superior glycemic control up to 5 years after surgery compared with intensive medical treatment alone^2,3^.

The mechanisms responsible for improving T2D glucose metabolism are still being debated, but early after surgery, the combined effects of caloric restriction and an exaggerated postprandial GLP-1 release, improving hepatic insulin sensitivity and beta-cell function, respectively, are among the most likely explanations^4^. Continued postprandial hypersecretion of anorexigenic gut-hormones limiting appetite and food intake, and leading to weight loss and increased peripheral insulin sensitivity, may explain later effects of RYGB on glucose metabolism^5–9^.

However, epidemiological reports have questioned the durability of surgery in some patients, and indicated that weight regain and deteriorating glycemic control may occur late after surgery^10,11^. Thus, 25–50% of patients with diabetes remission at 1 year will have relapsed 5 years after RYGB^12,13^, but determining physiological factors of glycemic control late after surgery is not well characterized.

In persons with normal glucose tolerance (NGT) before the operation, a late complication to RYGB is postprandial hypoglycemia^14^. This has been linked to exaggerated insulin release in response to rapid increases in postprandial glucose and GLP-1 concentrations^15,16^.

To address the development of these glycemic disturbances, we studied the effects of RYGB on glycemic control as well as measures of insulin sensitivity, beta-cell function and body weight late after RYGB surgery in patients with NGT or T2D prior to surgery, and results were compared to preoperative and 1-year (1 y) post RYGB results. Additionally, we describe how changes in these physiological parameters relate to changes in glycemic control in patients with T2D and with postprandial glucose nadir in subjects with NGT.

## Materials and Methods

### Subjects

Patients with NGT or T2D, who had participated in one of 3 previous studies (ClinicalTrials.gov ID: NCT00810823, NCT01993511, NCT01202526) evaluating the earlier effects of RYGB in response to an oral glucose stimulus, were invited to participate in a late follow-up, performed >2 years after surgery. *Cohort 1:* Subjects from two previous studies who received RYGB surgery >2 years prior to May 2013^6,17^; *Cohort* 2*:* subjects from a previous study studied 3.5–5 years after RYGB^9^. Preoperative glucose tolerance is defined in Table 1. Antidiabetic medications were discontinued 3 days (GLP-1 receptor agonists: ≥10 days) before each study day; all antidiabetic medications were discontinued from time of surgery. Before inclusion, all participants fulfilled the inclusion criteria for bariatric surgery in Denmark and had completed a preoperative diet-induced total body weight loss of at least 8% required by the Danish health authorities. All trial extensions were approved by the Danish Capital Region Municipal Ethical Committee and by the Danish Data Protection Agency and the study performed in accordance with the Helsinki-II declaration.

**Table 1:** Definition of preoperative glycemic control.

NGT	Fasting plasma glucose <6.1 mM *and* 2 hour OGTT plasma glucose <7.8 mM *and* HbA1c <42 mmol/mol
T2D	Fasting plasma glucose >7.0 mM *and/or* 2 hour OGTT plasma glucose >11.1 mM *and/or* Treatment with ≥1 antidiabetic agents

### Surgical procedure

Standard RYGB procedure as previously described^6^.

### Oral stimulation tests

Three fasting blood samples were drawn after an overnight fast (10 h), followed by a liquid mixed meal test in *Cohort 1* (200 ml Fresubin Energy Drink [300 kcal, carbohydrate (E% 50), protein (E% 15), fat (E% 35), Fresenius Kabi, Deutschland, Bad Homburg, Germany]) or an oral glucose tolerance test (OGTT) in *Cohort 2* (75 g anhydrous glucose dissolved in 250 ml of water). Following the oral stimulus (T = 0), blood was sampled frequently for 180 min in cohort 1 and for 120 min in cohort 2 (15, 30, 45, 60, 90, 120 min, and in Cohort 1 only, 180 min).

### Sample collection

Blood samples were collected in clot-activator tubes for C-peptide and insulin analyses, left to coagulate for 30 min, spun, aliquoted, frozen and stored at −80 C. Blood for glucose analysis was collected in prechilled EDTA-tubes, blood for GIP and GLP-1 measurements was collected in prechilled EDTA tubes containing a DPP-4 inhibitor (valine-pyrrolidide; 0.01 mmol/l, final concentration), immediately cooled on ice and centrifuged at 4 °C. Plasma for GIP and GLP-1 analysis were stored at −20 C.

### Laboratory analyses

Glucose was measured bedside using the glucose oxidase technique (YSI model 2300 STAT Plus, Yellow Spring Instruments, Yellow Spring, OH). Serum insulin and C-peptide concentrations were determined using AutoDELFIA fluoroimmunoassay, Wallac OY, Turku, Finland^6^, or the Immulite 2000 analyzer, Siemens Healthcare Diagnostics, Tarrytown, NY^9,17^) analyzed in one batch per cohort. Similarly, incretins were measured in one batch (e.g. including re-analysis of preoperative and 1-year samples) in Cohort 1 only, due to lack of plasma to reanalyze in cohort 2. For the same reasons, incretins were only analyzed in the fasting state and at one timepoint postprandially (T = 45 min for GLP-1 and T = 60 min for GIP). Total GLP-1 and GIP was analyzed as previously described^18^, using a radioimmunoassay (RIA, antiserum no. 89390) specific for the C-terminal of the GLP-1 molecule and reacting equally with intact GLP-1 and the primary (N-terminally truncated) metabolite; Total GIP was assayed using the C-terminally directed antiserum #867, which equally recognizes both intact GIP (1–42) and the primary metabolite, GIP (3–42)^19^.

### Calculations and statistical analyses

Data are expressed as means ± SE. Total area-under-the-curve (T-AUC) was calculated using the trapezoidal model. Excess body weight loss (EBWL) was calculated as (preoperative BMI − study BMI)/(preoperative BMI − 25) × 100%. HOMA2-IR C-peptide was calculated using the University of Oxford HOMA calculator (www.dtu.ox.ac.uk/homacalculator/download.php). Prehepatic insulin secretion rates (ISR) were calculated by deconvolution of peripheral C-peptide concentrations and application of population-based parameters for C-peptide kinetics using the ISEC software^20,21^ and expressed as pmol·kg^−1^·min^−1^. Beta-cell glucose sensitivity (bGS), i.e. increment in ISR in response to 1 mmol/L increase in plasma glucose, was calculated as previously described during the upslope of the glucose curve (meal start to peak glucose)^6^. Disposition index (DI) was calculated as bGS × insulin sensitivity (1/HOMA2-IR). Relative changes from 1 y to late after RYGB, were calculated as (relevant value_late_/relevant value_1y_ − 1)∙100%. Relative changes in weight, insulin resistance and beta-cell function, were related to the relative change in measures of glycemic control and nadir glucose using a linear regression model in the individual groups (NGT/T2D). The correlation coefficient was calculated using Pearsons correlation analysis for each group.

Weight loss failure was defined as EBWL less than 50%^22^. Diabetes remission was defined according to ADA criteria^23^.

Within group postoperative changes were analyzed with Wilcoxon’s matched pairs signed rank test, between group differences with Wilcoxon’s signed rank test. Comparison of proportions between groups were done using the Chi-squared test. A p-value < 0.05 was considered statistically significant. Calculations and statistical analyses were performed with the R statistical software (R version 3.3.2)^24^.

### Ethical statement

All procedures performed in studies involving human participants were in accordance with the ethical standards of the institutional and/or national research committee and with the 1964 Helsinki declaration and its later amendments or comparable ethical standards.

### Consent statement

Informed consent was obtained from all individual participants included in the study.

## Results

**Figure 1:**
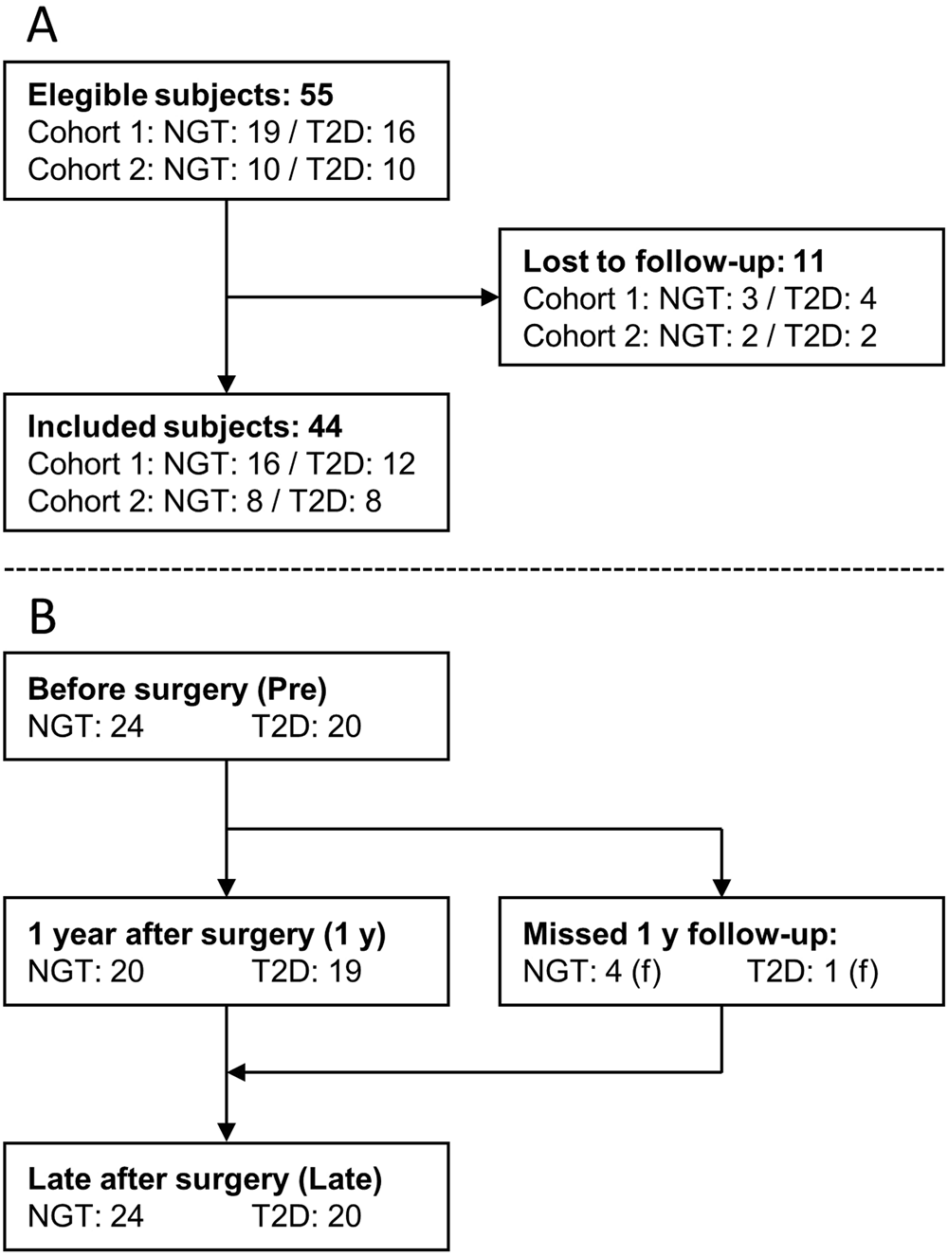
(**A**) Flow chart illustrating the inclusion of patients. (**B**) Flow of included patients in the study.

Of 55 eligible subjects, 44 accepted the invitation to the late follow-up (24 NGT, age: 44 ± 2 years/20 T2D, age: 49 ± 2 years, median follow-up 2.7 [Range 2.2–5.0 years] (Fig. 1). Patients lost to follow-up did not differ from participants with respect to preoperative age, bmi, hba1c or diabetes duration or with respect to 1 y postoperative EBWL (data not shown). 3 patients (2 T2D/1 NGT) in *cohort 2* refused the OGTT postoperatively due to a general distaste for the oral stimulus, previous problems with malfunctioning iv access and/or lack of time. These patients are represented with biometric and fasting data only. Preoperative antiglycemic treatment is shown in Table 2. All but two T2D patients, were without antidiabetic medication at the late follow-up. Patient characteristics are presented in Table 3.

**Table 2:** Preoperative antiglycemic treatment in patients with type 2 diabetes.

	Diet	MET	MET + SU	MET + LIRA	MET + SU + EX	MET + SU + TZD	MET + INS
Number	2	10	4	1	1	1	1

MET = Metformin, SU = Sulfonylurea, LIRA = Liraglutide, EX = Exenatide, TZD = Glitazone, INS = Insulin.

**Table 3:** Weight and biometrics.

	NGT			T2D		
	*Pre*	*1 y*	*Late*	*Pre*	*1 y*	*Late*
Number	24	20	24	20	19	20
Male gender	6	6	6	12	12	12
Time after surgery (weeks)	−0.5 [−1.8–0.4] ± 0.8	53 [52–55]-	143 [131–200] ± 11	−0.5 [−2.4–0.1]	54 [53–57]	139 [132–223]
BMI (kg/m^2^)	41.6 ± 0.9	31.4 ± 1.2**	31.0 ± 1.1**	40.7 ± 1.1	31.6 ± 1.3**	32.1 ± 1.4**
EBWL (%)	0 ± 0	67 ± 6**	68 ± 6**	0 ± 0	65 ± 6**	61 ± 8**
Waist (cm)	123 ± 2	99 ± 3**	96 ± 3**	128 ± 3	107 ± 3**	106 ± 4**
Hip (cm)	127 ± 2	105 ± 2**	107 ± 2**	120 ± 2^‡^	105 ± 2**	107 ± 2**
Waist-hip ratio	0.98 ± 0.02	0.94 ± 0.02*	0.89 ± 0.02**	1.07 ± 0.03	1.02 ± 0.03*	0.99 ± 0.03*

^*^p < 0.05 vs pre; **p < 0.001 vs pre; ^†^p < 0.05 vs 1 y; ^‡^p < 0.05 vs NGT at corresponding time point. All parameters are mean ± SEM, except time after surgery, which is Median [IQR].

### Weight loss and biometric data

Patients lost weight during the first year after surgery, but late after RYGB, weight was unchanged from that at 1 y (Table 3). While average BMI was still >30, both groups had a mean excess body weight loss (EBWL) of more than 60%. The proportion of participants with EBWL <50% at 1 y was 35% and 34% late after surgery. EBWL late after surgery was numerically lower in the T2D patients, but the proportion of patients with >50% EBWL was the same irrespective of diabetes status (NGT: 17/24; T2D: 12/20, p = 0.45). Weight change from 1 y to late follow-up did not differ between *cohort 1* and *2* (p = 0.11). As subjects lost weight, waist/hip ratio declined.

### Glucose metabolism

**Figure 2:**
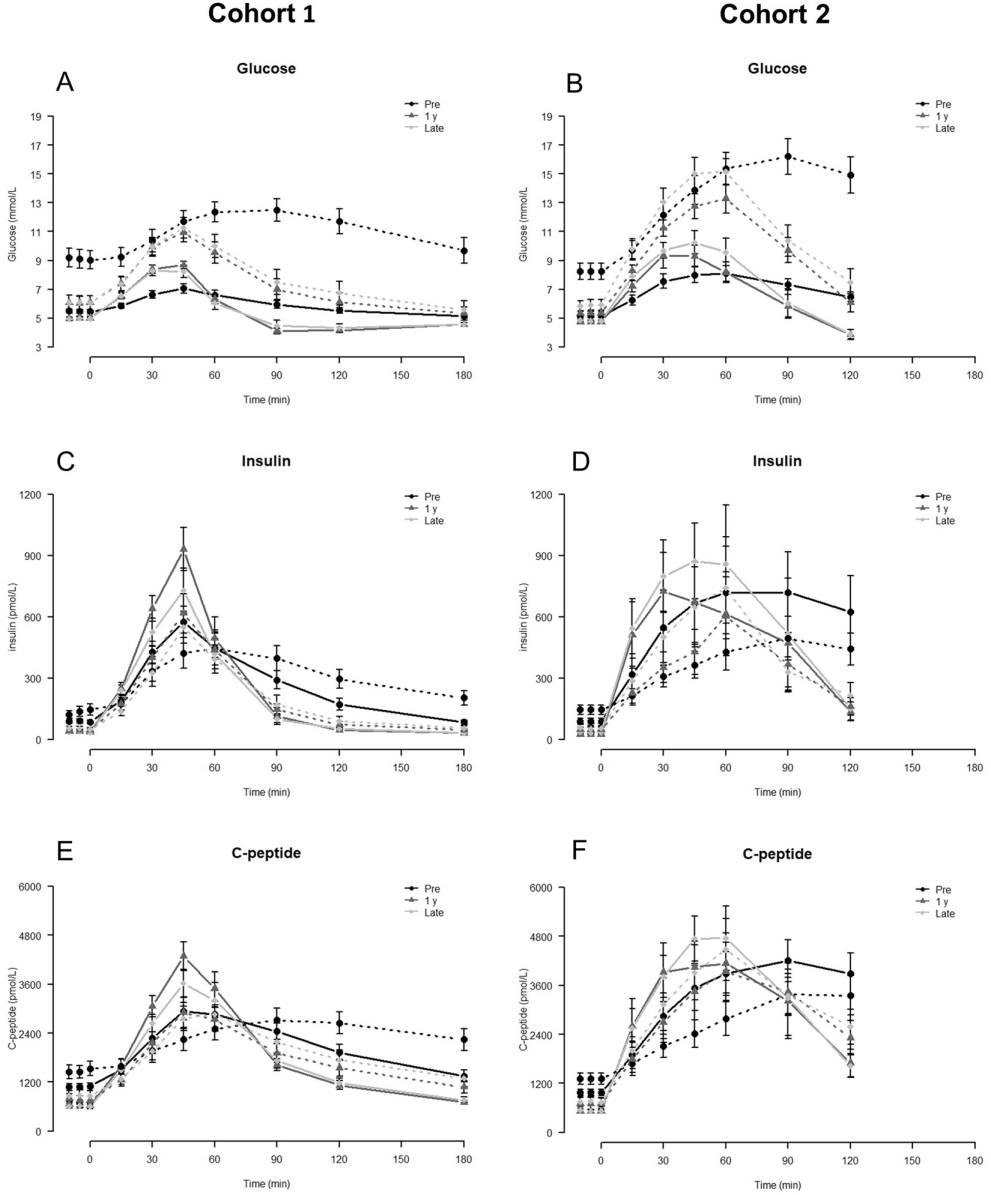
Glucose (A + B), Insulin (C + D) and C-peptide (E + F) concentrations in Cohort 1 (MMTT, A + C + E) and Cohort 2 (OGTT, B + D + F). Dotted lines are T2D, solid lines NGT. Data are mean ± SE.

In T2D patients, HbA1c was decreased late after surgery compared to before but had increased slightly compared to 1 y (Table 4). In both groups, fasting glucose concentrations remained unchanged late after surgery compared to 1 y, but incremental AUC glucose was only lower than preoperative values in T2D patients at 1y (Table 4 and Fig. 2). In T2D patients peak and nadir glucose values decreased after RYGB, whereas postprandial glucose excursions increased in NGT subjects after surgery with greater peak and lower nadir values (Table 4 and Fig. 2).

**Table 4:** Glycemic control, insulin and incretin secretion.

	NGT			T2D		
	Pre	1 y	Late	Pre	1 y	Late
**Glycemic control**						
HbA1c (mmol/mol)	36 ± 0.7	35 ± 0.7*	36 ± 0.9	52 ± 2^‡‡^	38 ± 1**^,‡^	42 ± 2**^,†,‡^
Fasting Glucose (mmol/L)	5.3 ± 0.1	4.9 ± 0.1**	4.9 ± 0.1**	8.8 ± 0.4^‡‡^	5.8 ± 0.3**^, ‡‡^	6.0 ± 0.3**^, ‡^
Peak Glucose (mmol/L)	7.6 ± 0.3	9.2 ± 0.3**	9.5 ± 0.4**	14.3 ± 0.8^‡‡^	11.9 ± 0.6*^,‡‡^	12.7 ± 0.7^‡‡^
Postprandial nadir glucose (mmol/L)	5.3 ± 0.2	3.9 ± 0.2**	3.8 ± 0.2**	9.2 ± 0.5^‡‡^	5.5 ± 0.3**^,‡‡^	6.2 ± 0.5**^,‡‡^
IAUC Glucose (mol × min/L)				‡‡	*, ‡‡	‡‡
Cohort 1 (0–180 min)	80 ± 21	81 ± 18	94 ± 40	367 ± 40	239 ± 26	301 ± 49
Cohort 2 (0–120 min)	244 ± 36	271 ± 58	331 ± 65	665 ± 74	564 ± 66	667 ± 63
**Insulin secretion**						
Fasting Insulin (pmol/L)	88 ± 8	34 ± 4**	36 ± 4**	139 ± 16‡	46 ± 7**	54 ± 7**
Peak Insulin (pmol/L)	671 ± 88	889 ± 94	868 ± 109	507 ± 62	625 ± 89*^,‡^	627 ± 94
IAUC Insulin (nmol × min/L)						
Cohort 1 (0–180 min)	32 ± 3	38 ± 5	32 ± 5	32 ± 5	29 ± 4	25 ± 4
Cohort 2 (0–120 min)	60 ± 19	56 ± 20	67 ± 22	28 ± 7	36 ± 11	45 ± 13
Fasting C-peptide (pmol/L)	1054 ± 56	597 ± 43**	588 ± 37**	1415 ± 111‡	746 ± 84**	822 ± 88**^,‡^
Peak C-peptide (pmol/L)	3620 ± 241	4434 ± 340*	4270 ± 364	3180 ± 263	3365 ± 339	3490 ± 364
IAUC C-peptide (nmol × min/L)		*		‡	**	**
Cohort 1 (0–180 min)	178 ± 14	223 ± 23	211 ± 23	157 ± 18	183 ± 19	180 ± 20
Cohort 2 (0–120 min)	286 ± 47	316 ± 71	341 ± 63	159 ± 30	264 ± 43	288 ± 48
Basal ISR (pmol × min^−1^ × kg^−1^)	2.6 ± 0.13	1.8 ± 0.11**	1.7 ± 0.10**	3.3 ± 0.22^‡^	2.1 ± 0.20**	2.2 ± 0.21**^, ‡^
IAUC ISR (pmol × kg^−1^)		*	*	‡	**	**
Cohort 1 (0–180 min)	510 ± 49	762 ± 103	676 ± 89	427 ± 46	582 ± 69	603 ± 78
Cohort 2 (0–120 min)	974 ± 149	1238 ± 279	1262 ± 247	528 ± 95	980 ± 140	1008 ± 202
**Incretins (Cohort 1)**						
Basal GLP-1 (pmol/L)	12 ± 1	9 ± 1	12 ± 1^†^	12 ± 1	10 ± 1	15 ± 2^†^
Postprandial GLP-1 (pmol/L), T = 45 min	15 ± 1	N/A	64 ± 10**	16 ± 2	N/A	72 ± 9**
Basal GIP (pmol/L)	7 ± 1	7 ± 1	8 ± 1	10 ± 2	9 ± 1	10 ± 1
Postprandial GIP (pmol/L), T = 60 min	68 ± 9	37 ± 3*	45 ± 5**	65 ± 4	46 ± 2*	52 ± 4*

^*^p < 0.05 vs pre, **p < 0.001 vs pre; ^†^p < 0.05 vs 1 y, ^††^p < 0.001 vs 1 y; ^‡^*p* < *0.05 vs NGT*, ^‡‡^*p* < *0.001 vs NGT* at corresponding time point.

### Insulin resistance, insulin secretion and beta-cell function

**Figure 3:**
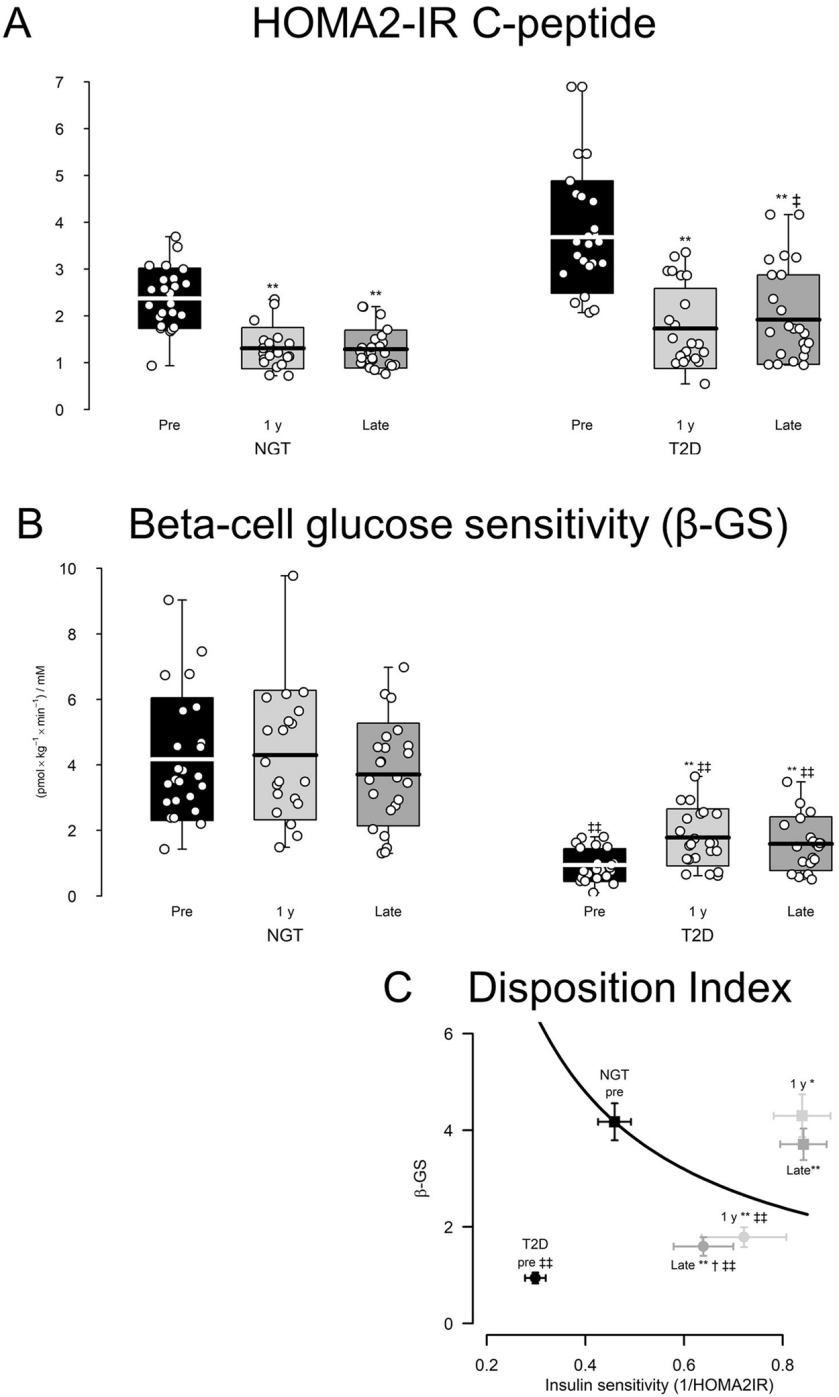
Effect of RYGB surgery on measures of glucose metabolism in T2D and NGT subjects. (**A,B**) Data are mean ± standard deviation, individual data points are indicated. Arrows indicate minimum and maximum data points. (**C**) Data are mean ± SEM. *p < 0.05 vs pre, **p < 0.001 vs pre; ^†^p < 0.05 vs 1 y, ^††^p < 0.001 vs 1 y; ^‡^p < 0.05 vs NGT, ^‡‡^p < 0.001 vs NGT at corresponding time point.

After surgery, insulin resistance (HOMA2-IR C-peptide) decreased in parallel in NGT and T2D subject and remained ~50% reduced at the late follow-up, but T2D patients were more insulin resistant than NGT subjects at all times (Fig. 3A). Basal prehepatic insulin secretion rate (basal ISR) decreased after RYGB in both groups whereas postprandial (IAUC) ISR increased (Table 4). The response was greater in the NGT than in the T2D group. In contrast, IAUC insulin did not significantly change in any of the groups. Late after surgery, bGS remained increased in T2D patients and unchanged in NGT subjects compared to before surgery (Fig. 3B). DI was 4-fold increased in T2D patients and 90% increased in NGT subjects late after compared to before RYGB (Fig. 3C), despite a slight reduction in DI late after compared to 1 y after surgery in patients with T2D.

### Incretins (cohort 1)

Basal GLP-1 and GIP concentrations remained unchanged late after RYGB surgery. Postprandial GIP concentrations (at 60 min) were decreased and postprandial GLP-1 secretion (at 45 min) markedly increased late after RYGB compared to presurgical levels (Table 4).

### Diabetes status late after RYGB

Six (Cohort 1: 4/Cohort 2: 2) of 20 T2D patients had HbA1c >48 mmol/mol (>6.5%) and/or fasting glucose >7 mmol/L late after RYGB, resulting in a 30% relapse rate late after RYGB. Patients with diabetes relapse did not differ from patients in remission with respect to age, EBWL or insulin resistance, but had poorer bGS (0.9 ± 0.2 vs 1.9 ± 0.2 pmol·kg^−1^·min^−1^, p = 0.004) and DI (0.42 ± 0.04 vs. 1.2 ± 0.2 pmol·kg^−1^·min^−1^, p = 0.010) at the late follow-up. Preoperatively, patients with diabetes relapse late after RYGB did not differ with respect to BMI or insulin resistance but had longer diabetes duration (8.3 ± 1.1 vs 2.9 ± 0.7 years, p = 0.004) and poorer beta-cell glucose sensitivity (0.6 ± 0.1 vs 1.1 ± 0.1 pmol·kg^−1^·min^−1^, p = 0.026) compared to late remitters.

### Correlation of measures of glucose metabolism to measures of glycemic control and glucose tolerance

**Figure 4:**
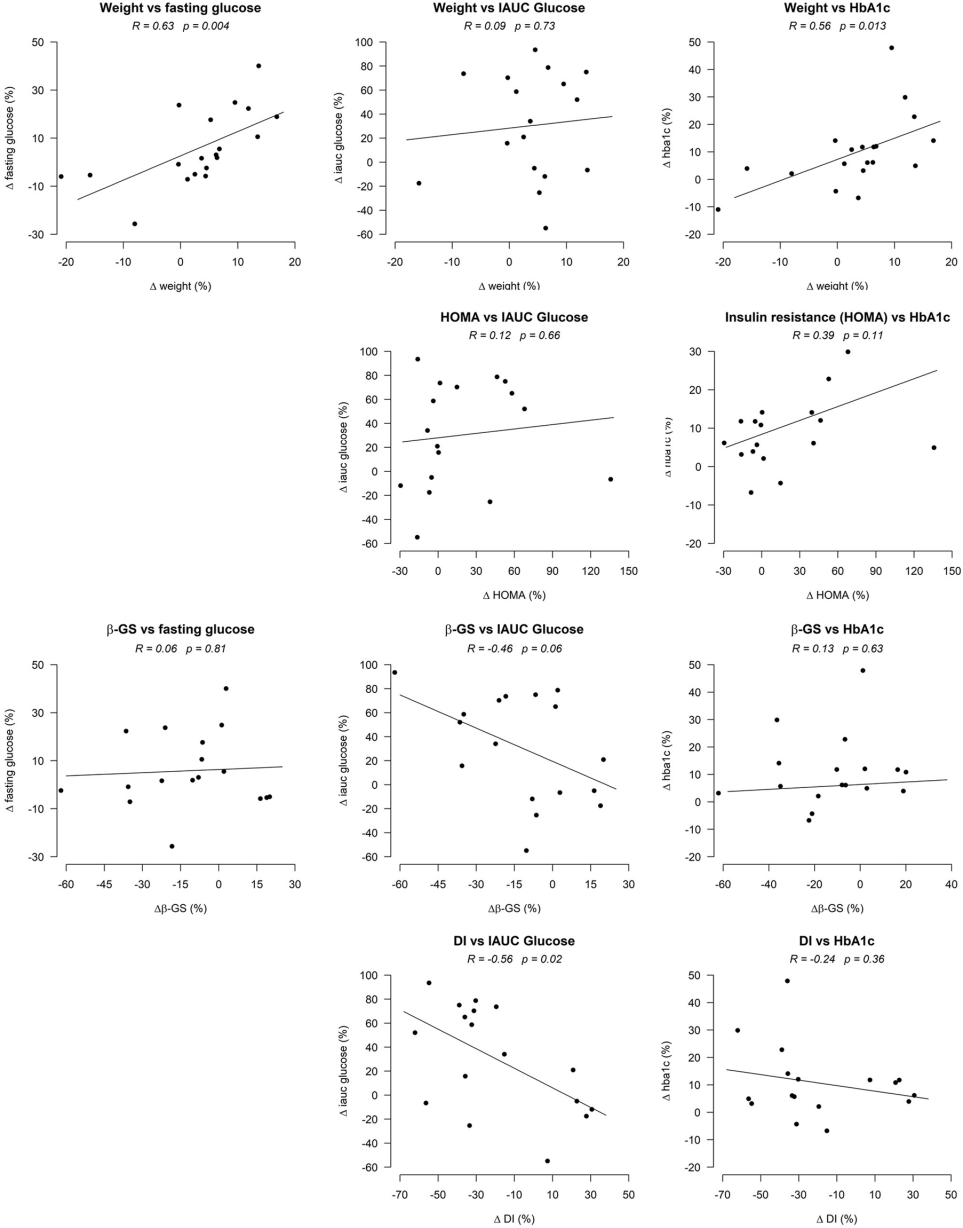
Correlation of relative changes in weight, insulin resistance (HOMA), beta-cell glucose sensitivity (bGS) and disposition index (DI) and changes in fasting and postprandial glucose and HbA1c in patients with type 2 diabetes from 1 y to late after surgery.

In T2D patients, the percentage (relative) weight change from 1 y to late after surgery were positively correlated with relative changes in fasting glucose concentrations and HbA1c, but not postprandial glucose excursions (Fig. 4). Relative changes in bGS and DI correlated inversely to changes in IAUC glucose.

In NGT subjects, relative changes in fasting glucose correlated with percent weight change (R = 0.71, p < 0.001). Small changes in HbA1c or postprandial glucose excursions were not associated with changes in body weight, insulin resistance or beta-cell function. Relative changes in postprandial nadir glucose in NGT subjects from 1 y to late post RYGB correlated negatively with bGS (R = −0.46, p = 0.041) but not with relative changes in peak C-peptide concentrations (R = −0.20, p = 0.40).

## Discussion

In this follow-up study, patients with T2D or NGT prior to surgery maintained a substantial weight loss with a lower waist-hip ratio 2.5–4 years after RYGB. Fasting glucose and HbA1c remained significantly reduced late after surgery in T2D patients compared to before the operation and, although postprandial glucose excursions (IAUC) did not differ at late follow-up, postprandial glucose concentrations were lower. In NGT subjects, glucose tolerance was unchanged and nadir glucose lower late after RYGB. In both groups, insulin resistance, remained reduced, and DI, a measure of beta-cell function, elevated late after surgery, while GLP-1 secretion remained increased compared to preoperative concentrations.

The weight loss reported here corresponds well with previous studies^1^, while the proportion of weight loss failures, ie EBWL <50%, accounting for 34% of the total population, was a little higher than expected^22,25^. This did not differ between patients with T2D or NGT and had not increased compared to 1 year after surgery.

Patients with preoperative T2D as a group experienced lasting beneficial effects on basic parameters of glucose metabolism after RYGB: insulin resistance remained diminished and beta-cell function improved late after surgery. These metabolic improvements undoubtedly explain the long-term superiority of RYGB surgery compared to intensive medical treatment alone^2,26,27^.

We and others have previously shown how the improved beta-cell function after RYGB is highly reliant on the exaggerated postprandial GLP-1 secretion^28–30^, and indeed we found that postprandial GLP-1 concentrations were elevated late after RYGB as previously reported earlier after surgery, indicating that the capacity of the gut to hypersecrete GLP-1 and the stimulatory effect of GLP-1 on beta-cells does not wane^9,17,31^. However, this prolonged GLP-1 stimulation in RYGB patients does not lead to a further improvement in beta-cell function either. GIP secretion appeared attenuated late after RYGB surgery, but this finding must be interpreted cautiously because of the limited postprandial samples.

T2D patients may experience weight regain and deteriorating glucose tolerance late after surgery^10,11^. In this study, patients who had diabetes relapse were characterized by longer preoperative diabetes duration, which is in accordance with previous findings^32^, and poorer beta-cell function both before and after surgery compared with patients who remained in remission late after RYGB. This stresses the importance of beta-cell function in T2D glucose metabolism^33^. It is important to recognize that patients in this study still had diabetes immediately pre-surgery after completion of a required pre-operative 8% weight loss, which substantially improves glucose metabolism^34^. Thus, by design, our study, selected patients with more severe diabetes, and in this context a remission rate of 70% is impressive.

Our correlation analyses show that maintenance of weight loss and postoperative improvements in beta-cell function are important for persisting improvements in post RYGB glycemic control in patients with preoperative T2D. Since postprandial GLP-1 hypersecretion after RYGB causes both improved beta-cell function, and suppresses appetite and decreases food intake together with other hypersecreted anorexigenic gut hormone, these findings support that GLP-1 plays a central role for the beneficial effects of this surgery^8,35–37^.

In patients with preoperative NGT, we demonstrated an association between changes in fasting glucose and weight loss after surgery, which is to be expected, and a negative correlation between nadir glucose and bGS. The latter association is mechanistically sound, and could reflect insulinotropic effects of the exaggerated postprandial GLP-1 release^16,28,38^. However, an association could not be shown between changes in peak C-peptide concentrations, another measure of beta-cell secretory capacity, and nadir glucose concentrations, possibly reflecting the importance of early insulin secretion for glucose tolerance^39^.

Only 80% of eligible subjects participated in the late follow-up session, introducing the risk of selection bias, but dropouts did not differ from participants with respect to basic preoperative and 1 y postoperative characteristics. Another limitation is the use of two different oral stimuli, but since participants were their own controls and we defined diabetes relapse from fasting glucose and HbA1c, the impact on results was limited^40^. Further, the 120 min follow-up during the OGTT in cohort 2, may have led to nadir glucose being missed in some patients. Finally, shortage of extra plasma for re-analysis limited our incretin data to one fasting and one postprandial timepoint in Cohort 1 only.

In conclusion, we find that RYGB surgery provides a sustained weight loss 2.5–4 years after RYGB surgery in morbidly obese patients with and without preoperative T2D, and that the beneficial effects on glucose metabolism seen 1 year after the operation are also present late after surgery in most patients. Maintenance of weight loss and improved beta-cell function is important for postoperative glycemic control in patients with preoperative T2D, while the increased insulin secretion in response to glucose may explain postprandial hypoglycemia in NGT subjects after RYGB.
